# Anterior Segment Findings in Women with Polycystic Ovary Syndrome

**DOI:** 10.4274/tjo.73659

**Published:** 2017-01-17

**Authors:** Seda Karaca Adıyeke, Ibrahim Karaca, Suna Yıldırım, Mehmet Adıyeke, İbrahim Uyar, Gamze Türe

**Affiliations:** 1 Tepecik Training and Research Hospital, Ophthalmology Clinic, İzmir, Turkey; 2 Tepecik Training and Research Hospital, Gynecology and Obstetrics Clinic, İzmir, Turkey; 3 Aliağa State Hospital, Gynecology and Obstetrics Clinic, İzmir, Turkey; 4 Bergama State Hospital, Gynecology and Obstetrics Clinic, İzmir, Turkey

**Keywords:** Polycystic ovary syndrome, Dry eye, corneal thickness

## Abstract

**Objectives:**

This study aimed to investigate the anterior segment in women with polycystic ovary syndrome (PCOS) and to compare them with those of healthy reproductive-age female volunteers.

**Materials and Methods:**

The study included 50 right eyes of 50 women with PCOS (group 1) and 50 right eyes of 50 healthy women (group 2). Intraocular pressure, Schirmer’s test, tear film break-up time and central corneal thickness were evaluated in all subjects. Correlations between serum hormone (estradiol and testosterone) levels and observed findings were also investigated.

**Results:**

Mean central corneal thickness values were significantly higher in the PCOS group (p=0.001). The mean intraocular pressures values were similar between the two groups (p=0.560). Schirmer’s test results and tear film break-up time values were significantly lower in the PCOS group (p=0.001 and p=0.001 respectively). Serum estradiol levels were moderately positively correlated with mean central corneal thickness (r=0.552), weakly positively correlated with intraocular pressure (r=0.351) and weakly negatively correlated with tear film break-up time (r=-0.393). Serum free testosterone levels were weakly correlated with intraocular pressure (r=0.342) and central corneal thickness (r=0.303), and showed weak negative correlations with Schirmer’s test results (r=-0.562) and tear film break-up time (r=-0.502).

**Conclusion:**

PCOS leads to physiological and structural changes in the eye. Dry eye symptoms were more severe and central corneal thickness measurements were greater in patients with PCOS. Those are correlated serum testosterone and estradiol levels.

## INTRODUCTION

Polycystic ovary syndrome (PCOS) is the most common endocrinopathy among women of reproductive age. The prevalence is generally about 17%, though the rate varies with different diagnostic criteria.^1^ Chronic anovulation seems to be the main physiopathologic factor.^1^ It is characterized by elevated circulating levels of the most biologically active androgens secreted primarily by the ovaries, such as androstenedione and testosterone.

In recent years it has been discovered that female sex steroids have both systemic and ocular effects. Estrogen, progesterone and androgen receptors have been found in the cornea, lens, iris, ciliary body, retina, lacrimal glands, meibomian glands and conjunctiva.^2^ This is best illustrated by the fact that dry eye is more prevalent among women, especially following menopause, increases during pregnancy and lactation, and resolves with hormone replacement therapy.^3^ In the present study, we aimed to compare central corneal thickness (CCT), intraocular pressure (IOP), tear film break-up time (TBUT) and Schirmer test values between PCOS patients and healthy individuals. We evaluated the correlation between these findings and testosterone and estradiol levels and body mass index.

## MATERIALS AND METHODS

The study was approved by the Turkish Ministry of Health Public Hospitals Administration, General Secretary of the Association of Public Hospitals in the Northern İzmir Province, Tepecik Training and Research Hospital Local Ethics Committee. Informed consent was obtained from all study subjects. The study included 50 women who presented to the Tepecik Training and Research Hospital and were diagnosed with PCOS between September 2014 and November 2014, and 50 female volunteers with no endocrine-related complaints or disorders who presented to our clinic during the same period. The study was conducted in accordance with the principles stated in the Declaration of Helsinki. As per the Rotterdam 2003 criteria, PCOS diagnosis was based on the presence of at least 2 of the following: oligomenorrhea (more than 45 days between menstrual periods or fewer than 8 menstrual periods per year), hyperandrogenism, clinical hirsutism (acne, hirsutism, androgenic alopecia, acanthosis nigricans) or laboratory findings indicating elevated androgen levels (elevated serum total or free testosterone levels), and the appearance of polycystic ovary on ultrasonography (2-9 mm in diameter, 12 or more follicles and/or increased ovary volume [>10 mL]).^4^ Thyroid functions, luteinizing hormone (LH)/follicle-stimulating hormone (FSH) ratio, and prolactin, dehydroepiandrosterone sulfate, 17-hydroxyprogesterone, and total testosterone levels were assessed for all patients. Samples were taken from all patients at the same time of day. Patients with thyroid disease, hyperprolactinemia, Cushing’s syndrome, or congenital adrenal hyperplasia, and patients who used drugs such as hormonal drugs, ovulation-inducing agents, glucocorticosteroids, or antiandrogens within the previous 6 months were not included in the study. Patients with optic neuropathy, retinal disease which may affect visual field or the retinal nerve fiber layer, or history of ocular surgery, severe ocular trauma, intracranial lesion, head trauma, massive blood loss, or contact lens use were not included in the study. A detailed medical history was obtained from the study participants and demographic data such as their age, personal history and family history were recorded. All subjects underwent a standard ophthalmologic examination. IOP was measured using a Goldmann applanation tonometer and CCT was measured using a non-contact specular biomicroscope. The average of 3 consecutive measurements varying less than 10 μm was recorded as the CCT value.

All patients underwent TBUT and Schirmer tests. The Schirmer test was performed under topical anesthesia. Wetting less than 6 mm in 5 minutes was considered abnormal. The tear film was examined at the slit-lamp under cobalt-blue filter using a wide beam. TBUT was evaluated as the interval between last blink and the first appearance of a dry spot in any area of the ocular surface. A TBUT of less than 10 s was considered abnormal.

Venous blood samples were collected from participants in the early follicular phase between the 3^rd^ and 5^th^ days of a spontaneous or gestagen-induced menstrual cycle. Venous blood was collected from the forearm between 8:00 and 10:00 a.m. following a 12-hour fast. LH, FSH, estradiol, and total testosterone measurements were done in the biochemistry laboratory of the Tepecik Training ve Research Hospital. Correlation analysis was performed between the serum estradiol and testosterone levels of PCOS patients and their Schirmer test scores, TBUT, mean CCT and IOP values. Data were statistically analyzed using SPSS version 15.0 (SPSS Inc., Chicago, IL, USA) software package. Pearson correlation analysis was used to assess relationships between variables. The normality of variable distributions was assessed. Continuous variables were presented as median, minimum and maximum values. Normally distributed independent variables not suitable for a t-test were compared using the Mann-Whitney U test. Intergroup differences with p values less than 0.05 were accepted as statistically significant.

## RESULTS

The study included a total of 100 right eyes from 50 patients in the PCOS group and 50 patients in the control group. The mean age was 27±4.18 years (range, 19-30 years) in the PCOS group and 26.4±3.78 years (range, 18-30 years) in the control group. There was no significant difference in age between the two groups (p=0.34).

**Figure 1:**
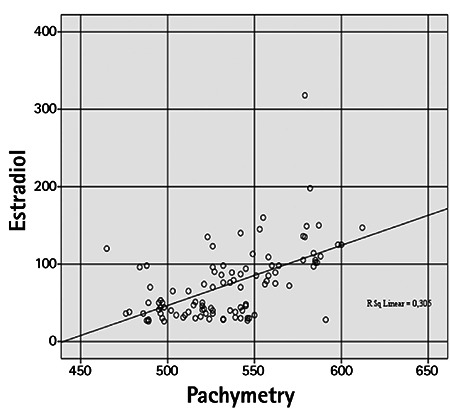
Correlation between serum estradiol level (pg/mL) and central corneal thickness (µm)

**Figure 2:**
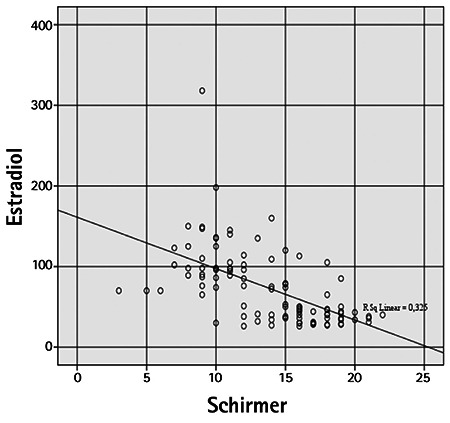
Correlation between serum estradiol level (pg/mL) and Schirmer test distance (mm)

**Figure 3:**
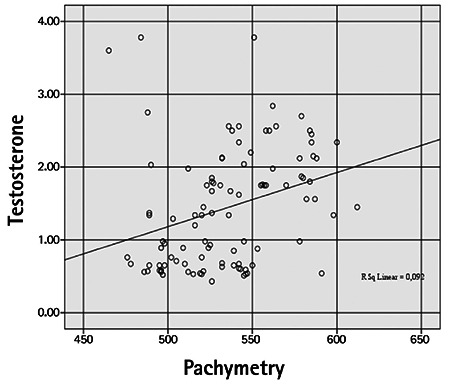
Correlation between serum testosterone level (pg/mL) and central corneal thickness (µm)

**Figure 4:**
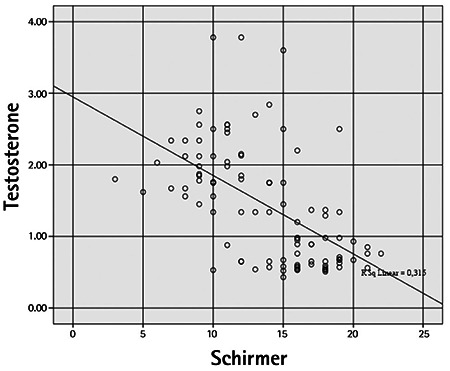
Correlation between serum testosterone level (pg/mL) and Schirmer test distance (mm)

Mean CCT was 550.60±32.38 µm in the PCOS group and 518.40±24.77 µm in the control group, which was a significant difference (p=0.001). Mean IOP values in the PCOS and control groups were 16.16±2.68 mmHg and 15.62±3.41 mmHg, respectively (p=0.56). The PCOS group had a significantly lower mean Schirmer test value compared to the control group (10.80±3.14 mm vs 16.74±2.61 mm; p=0.001). TBUT was 11.10±3.28 s in the PCOS group and 14.64±2.81 s in the control group. The PCOS group had a significantly shorter TBUT than the control group (p=0.001). In correlation analysis between estradiol and testosterone levels of PCOS patients and the ocular variables, estradiol showed a weak positive correlation with IOP (r=0.351) and a weak negative correlation with TBUT (r=-0.393). A moderate correlation was detected between serum estradiol level and CCT (r=0.552) (Figures 1, 2, 3 and 4). Weak correlations emerged between serum testosterone level and IOP (r=0.342) and CCT (r=0.303) (Figure 3). There was a moderate negative correlation between testosterone level and Schirmer test distance (r=-0.562) and TBUT (r=-0.494) (Figure 4).

## DISCUSSION

PCOS is the most common endocrinopathy in reproductive-age women, and is also referred to as ovarian hyperandrogenemia.^4^ The hyperestrogenemic effect cannot be balanced by progesterone and induces changes in the target organs. Ogueta et al.^5^ described estrogen-induced proteins (e.g. cathepsin D, alpha-2-macroglobulin, and aromatase cytochrome P45) which play an important role in vital cellular functions like differentiation, proliferation and maturation. Most of these proteins are found in ocular tissues such as the ciliary body and the retinal pigment epithelium.^5^ As in the cardiovascular system and endometrium, elevated sex steroid levels in PCOS also affect ocular structures and physiology.^6^ Steroid hormones play an important role in cellular processes like proliferation, differentiation and growth.^5^ Magness et al.^7^ found that estradiol is a potent vasodilator, while Sarrel^8^ reported that progesterone has the opposite effect. Yucel et al.^9^ proposed that low estrogen and high progesterone levels may have a vasoconstrictive effect that can reduce ocular perfusion.

Therefore, in the present study we compared tear function, IOP, and CCT in PCOS patients and healthy women. We analyzed the correlations between ocular findings and levels of free testosterone and estradiol.

Previous studies have reported that hormonal effects may cause aqueous layer deficiency and evaporative dry eye disease.^10,11,12,13,14^ Meibomian glands are a target organ of androgen hormones, which have been shown to regulate gene expression and lipid synthesis in these tissues.^10,11,12^ Androgen deficiency may lead to meibomian gland dysfunction and evaporative dry eye syndrome. Estrogen is an antagonist of meibomian gland function and may promote the development of evaporative dry eye.^13,14^ Na et al.^15^ documented substantial increases in dry eye syndrome and IOP in postmenopausal women using exogenic estrogen. IOP elevation was thought to be associated with the steroid effect of estrogen replacement therapy. Consistent with previous studies, the PCOS group in the current study showed significantly lower Schirmer test and TBUT values compared to the control group. Elevated serum estradiol and testosterone levels are correlated with dry eye findings. The strongest correlation was the negative correlation between estradiol levels and Schirmer test distance. Akar et al.^16^ reported that the neuroretinal rim showed significant thinning during the luteal phase, though no significant differences emerged in IOP, keratometry or refractive error when compared with the menstrual phases. A study by Demir et al.^17^ revealed comparable IOP and CCT values between PCOS patients and healthy subjects. In the current study, no significant difference in IOP values was detected between the PCOS and control groups. A weak correlation was observed between IOP and both free testosterone and estradiol levels. In a study by Kebapcılar et al.,^18^ PCOS patients exhibited significantly greater CCT bilaterally. The authors reported that CCT in both eyes was positively correlated with total testosterone levels, body mass index, insulin and insulin-like growth factor-1 (IGF-1). They attributed the significantly greater CCT values seen in PCOS patients to IGF-1 inhibition of the corneal endothelial pump and increased endothelial permeability.

Kiely et al.^19^ found that corneal thickness increased in association with rising estrogen levels during the menstrual cycle. In the current study, CCT was also significantly higher in the PCOS group than in the control group. Pachymetry values were weakly correlated with testosterone levels and moderately correlated with estradiol levels.

### Study Limitations

Limitation of this study are the variable repeatability and reliability of the Schirmer and TBUT tests, a relatively short follow-up period and a study design that was not prospective. Due to the large patient number and hormonal changes, a longer follow-up period is required.

## CONCLUSION

Our PCOS patients had significantly greater CCT and higher rates of tear film dysfunction compared to the control group. In light of these data, for PCOS patients it is advisable to plan corneal surgical procedures after hormone regulation has been achieved with medical therapy.

### Ethics

Ethics Committee Approval: The study was approved by the Turkish Ministry of Health Public Hospitals Administration, General Secretary of the Association of Public Hospitals in the Northern İzmir Province, Tepecik Training and Research Hospital Local Ethics Committee, Informed Consent: Informed consent was obtained from all study subjects.

Peer-review: Externally peer-reviewed.
